# Development of a deep learning model that predicts Bi-level positive airway pressure failure

**DOI:** 10.1038/s41598-022-12984-x

**Published:** 2022-05-26

**Authors:** Daniel D. Im, Eugene Laksana, David R. Ledbetter, Melissa D. Aczon, Robinder G. Khemani, Randall C. Wetzel

**Affiliations:** 1 Department of Pediatrics, Keck School of Medicine, University of Southern California, 2020 Zonal Ave, IRD 114, Los Angeles, CA 90089 USA; 2 Department of Anesthesiology and Critical Care Medicine, Children’s Hospital Los Angeles, Los Angeles, CA USA; 3 Laura P. and Leland K. Whittier Virtual Pediatric Intensive Care Unit, Children’s Hospital Los Angeles, Los Angeles, CA USA; 4 Department of Anesthesiology, Keck School of Medicine, University of Southern California, Los Angeles, CA USA

**Keywords:** Diagnosis, Paediatrics, Respiratory signs and symptoms, Respiratory tract diseases, Paediatric research

## Abstract

Delaying intubation for patients failing Bi-Level Positive Airway Pressure (BIPAP) may be associated with harm. The objective of this study was to develop a deep learning model capable of aiding clinical decision making by predicting Bi-Level Positive Airway Pressure (BIPAP) failure. This was a retrospective cohort study in a tertiary pediatric intensive care unit (PICU) between 2010 and 2020. Three machine learning models were developed to predict BIPAP failure: two logistic regression models and one deep learning model, a recurrent neural network with a Long Short-Term Memory (LSTM-RNN) architecture. Model performance was evaluated in a holdout test set. 175 (27.7%) of 630 total BIPAP sessions were BIPAP failures. Patients in the BIPAP failure group were on BIPAP for a median of 32.8 (9.2–91.3) hours prior to intubation. Late BIPAP failure (intubation after using BIPAP > 24 h) patients had fewer 28-day Ventilator Free Days (13.40 [0.68–20.96]), longer ICU length of stay and more post-extubation BIPAP days compared to those who were intubated ≤ 24 h from BIPAP initiation. An AUROC above 0.5 indicates that a model has extracted new information, potentially valuable to the clinical team, about BIPAP failure. Within 6 h of BIPAP initiation, the LSTM-RNN model predicted which patients were likely to fail BIPAP with an AUROC of 0.81 (0.80, 0.82), superior to all other models. Within 6 h of BIPAP initiation, the LSTM-RNN model would identify nearly 80% of BIPAP failures with a 50% false alarm rate, equal to an NNA of 2. In conclusion, a deep learning method using readily available data from the electronic health record can identify which patients on BIPAP are likely to fail with good discrimination, oftentimes days before they are intubated in usual practice.

## Introduction

Bi-level Positive Airway Pressure (BIPAP) is a form of non-invasive ventilation (NIV) increasingly used in adults and children with acute respiratory failure^1–3^. BIPAP can assist in lung recruitment, offload respiratory muscle work, and improve gas exchange^4^. BIPAP is an alternative to endotracheal intubation in many circumstances, although BIPAP failure frequently occurs, particularly in patients with hypoxemic respiratory failure and lung injury^5^.

Evidence is accumulating that delaying intubation for patients who are failing BIPAP is associated with harm, including prolonged ICU stay, increased rates of ventilator associated pneumonia and septic complications, and increased ICU mortality^6–11^. Potential mechanisms for this include Patient-Self Inflicted Lung Injury (P-SILI) from generation of high transpulmonary pressures, tidal volumes or atelectrauma with high respiratory drive; or higher rates of complications during intubation including desaturation and hypotension^12–21^. Therefore, identification of those likely to fail BIPAP may enable timely escalation to invasive mechanical ventilation and potentially prevent these complications.

Currently, the decision to intubate a child on BIPAP is primarily driven by clinical judgment. In children, severity of oxygenation abnormalities (such as the SpO2/FiO2) are also associated with intubation risk, although these metrics are neither sensitive nor specific^22,23^. For adults, the HACOR scale was developed to predict NIV failure in hypoxemic patients by comparing measurements of five variables – heart rate, pH, Glasgow Coma Score (GCS), PaO2/FiO2 ratio, and respiratory rate—to reference values^24^. Electronic Medical Records (EMR) and advanced machine learning (ML) methods provide an opportunity for timely and accurate identification of children likely to fail BIPAP. ML models can incorporate hundreds of variables, and deep learning neural networks, a subset of ML algorithms, can be trained to predict temporally evolving targets, including a patient’s medical status^25–30^. The objective of this study was to develop a deep learning neural network model capable of continuously predicting BIPAP failure following BIPAP initiation in critically ill children and compare it to multivariate logistic regression models using data available in the EMR. Additional objectives were to determine the characteristics of patients who fail BIPAP and investigate the impact of the timing of BIPAP failure on outcomes including Ventilator-free Days (VFDs), ICU length of stay (LOS), and mortality.

## Material and methods

### Data sources and variables

The data were extracted from anonymized clinical observations collected in Electronic Medical Records (EMR, Cerner) of children admitted to the Pediatric Intensive Care Unit (PICU) of Children’s Hospital Los Angeles between 2010 and 2020. EMR data for each episode included charted time series measurements for variables representing vitals, laboratory results, and medications and interventions. Data previously collected for Virtual Pediatric Services, LLC, including patient disposition, diagnoses, and PRISM-III scores, were linked with the EMR data before de-identification^27,31^. The research was approved by the CHLA Institutional Review Board. The study was conducted in accordance with the Declaration of Helsinki. The requirement to obtain informed consent from patients was waived by the IRB, as it was deemed non-humans subjects research.

### Cohort selection

Patient encounters where patients received BIPAP were included in this study. All BIPAP sessions of patients who had diagnoses of dependence on respirator/ventilator or obstructive sleep apnea were excluded because of the possibility of BIPAP dependence. Also excluded were BIPAP sessions of any patient who met at least one of the following criteria: (1) documented Do Not Resuscitate (DNR) or Do Not Intubate (DNI) order, (2) died while on BIPAP without intubation, given the likelihood of DNR or DNI status, (3) was transferred to another hospital while on BIPAP.

### Defining BIPAP failures and outcomes

*BIPAP Failure* was defined as*:* Escalation from BIPAP to endotracheal intubation with mechanical ventilation within 48 h of BIPAP termination.

*Early, Intermediate, and Late BIPAP Failure:* In this study, early BIPAP failure was defined as intubation within 6 h of BIPAP initiation. Intermediate BIPAP failure was intubation 6 to 24 h after BIPAP initiation. Late BIPAP failure was intubation more than 24 h after BIPAP initiation.

The number of *Ventilator-free Days (VFD)* at 28 days was calculated two ways: 28-VFD Invasive Mechanical Ventilation (IMV) was defined as the number of days within a 28-day time frame after IMV initiation that the patient was alive and not on IMV. Successful extubation from IMV was defined as not requiring re-intubation within 48 h of extubation^32,33^. 28-VFD IMV BIPAP was defined as the number of days within a 28-day time frame after BIPAP initiation that the patient was alive and neither on IMV nor BIPAP. Successful weaning from BIPAP was defined as not requiring BIPAP or IMV within 48 h of BIPAP termination. All patients with greater than or equal to 28 ventilator days and those who died within 28 days of mechanical ventilation were assigned 0 VFD (Examples of VFD calculation can be found in Supplementary Fig. 1).

### Statistical analyses to characterize BIPAP failures

The non-parametric Mann–Whitney U test was used to compare demographic variables and characteristics—vital signs, laboratory results, pediatric chronic complex conditions diagnoses (based on ICD-10) – between BIPAP failures and non-failures. Patients who met the early, intermediate, or late BIPAP failure definitions were characterized and compared in terms of their 28-VFD and 28-VFD IMV BIPAP. Post-extubation BIPAP and ICU LOS were also evaluated as outcome measures. The non-parametric Kruskal–Wallis H test was used for the comparisons among the three BIPAP failure groups. All statistical analyses were completed using Sci.Py 1.4.1 in Python 3.7.4.

### Data preprocessing

EMR measurements are asynchronously and irregularly charted. Pre-processing techniques described in previous work converted these measurements and other patient data into a matrix format amenable to machine learning. At any time when at least one variable had a recorded value, the missing values for other variables were imputed, and the process followed that of prior work^26,27^. Missing drug or intervention measurements were imputed with zero to indicate absence of treatment. When a physiologic observation or lab measurement was available, it was propagated forward until another measurement was recorded. When prior measurements were not available, the variable was imputed using the training set population mean. The S/F ratio, defined as SpO_2_ over FiO_2_, was used when SpO_2_ was between 80 and 97% as a measure of a patient's oxygenation^34^. FiO_2_ was treated the same regardless of BIPAP mask interface (nasal, oro-nasal, and total face mask). At any time when SpO_2_ fell outside of the interval (80%, 97%), the S/F ratio was forward filled from the last validly computed S/F ratio. Additional details can be found in Supplementary Fig. 2.

### Model development for predicting BIPAP failure

Prior to model development, the cohort of BIPAP sessions was partitioned into a training (80%) and a test (holdout) set (20%) for assessing model performance. Partitioning was done such that all BIPAP sessions of a single patient belonged to one of the sets. No other stratifications were applied. Three machine learning models were developed to predict BIPAP failure each time a new set of observations became available: two logistic regression models and a deep learning model—a recurrent neural network with a Long Short-Term Memory (LSTM-RNN) architecture^25–27^. The SpO_2_/FiO_2_ (S/F) ratio was evaluated as a reference model because it has been described as a useful outcome predictor in children as early as 1 h after BIPAP initiation^23^. The inputs to the first logistic regression model (LR_HACOR_) included four of the five HACOR scale input variables: heart rate, pH, GCS, and respiratory rate^24^. The pH values for LR_HACOR_ came from the arterial, capillary, and venous blood gas (ABG, CBG, VBG) measurements. The fifth HACOR scale variable, PaO2/FiO2 ratio, was replaced in LA_HACOR_ by the S/F ratio, a reliable noninvasive surrogate of the PaO2/FiO2 ratio, because SpO2 was more frequently available than PaO2^23,34^. Finally, SpO2 was also an LR_HACOR_ input. The second logistic regression model (LR_EMR_) used 301 variables representing vital signs, laboratory results, medications (antibiotics, vasopressors, inotropes, diuretics, sedatives, etc.), respiratory support (supplemental oxygen, BIPAP and ventilator settings), invasive procedures, radiography, and nursing assessments (see Supplementary Table 1 and Supplementary Table 2). It has been previously demonstrated that extraneous features from the EMR does not degrade LSTM-RNN performance^35^. The LSTM-RNN model has feedback/forward connections that allow it to process time series data in a sequential manner and integrate information from previous times with newly available inputs to inform current predictions. The LSTM-RNN model used the same 301 input variables as the LR_EMR_ (Supplementary Fig. 3 illustrates the flow of inputs into and outputs from the models, Supplementary Table 3 shows the hyperparameters of the LSTM-RNN). The two LR models were developed as comparators for the LSTM-RNN model, with LR_EMR_ serving as a bridge between LR_HACOR_ and the LSTM-RNN model.

Python 3.7.4, Scikit-learn 0.21.3, Keras 2.3.1, and Tensorflow version 2.1.0 were used to implement and train the LSTM-RNN and LR models to make a prediction each time there was a new observation in the data. Their parameters were derived from the training set.

### Model evaluation

#### Predicting BIPAP failure

Models were evaluated and compared for performance using the area under the receiver operating characteristic curve (AUROC). AUROC was computed for predictions at various hours (1, 2, …, 24) after BIPAP initiation (*n*-hour AUROCs). Performance was reported by bootstrap sampling the evaluation set 100 times, with each bootstrap iteration randomly selecting 75% of the BIPAP sessions without replacement in the set and calculating the AUROC of model predictions in that draw. The mean and 95% confidence interval of the AUROC scores were then calculated to report average performance and estimate population variance.

In the *rolling cohort n*-hour AUROCs, failures or successes that already occurred before the next hour of evaluation were excluded from the computation; thus, the number of cases used in the AUROC computation decreased over time. Number needed to alert (NNA)^36^ was also used to evaluate the predictions at 6 and 24 h after BIPAP initiation. NNA is defined as the sum of true positives and false positives divided by the number of true positives for any specific threshold. Note that NNA is the inverse of positive predictive value.

#### Subgroup analysis: hypoxemic patients

Model performance in patients with a low S/F ratio at the time of 6-h and 24-h predictions were evaluated to determine whether the model better discriminates BIPAP failure in hypoxemic patients than in non-hypoxemic patients. S/F ratio was considered low when it was less than 264, which is a diagnostic criterion for Pediatric Acute Respiratory Distress Syndrome (PARDS) for patients on NIV^37^.

## Results

### Demographics and characteristics of BIPAP cohort

**Figure 1:**
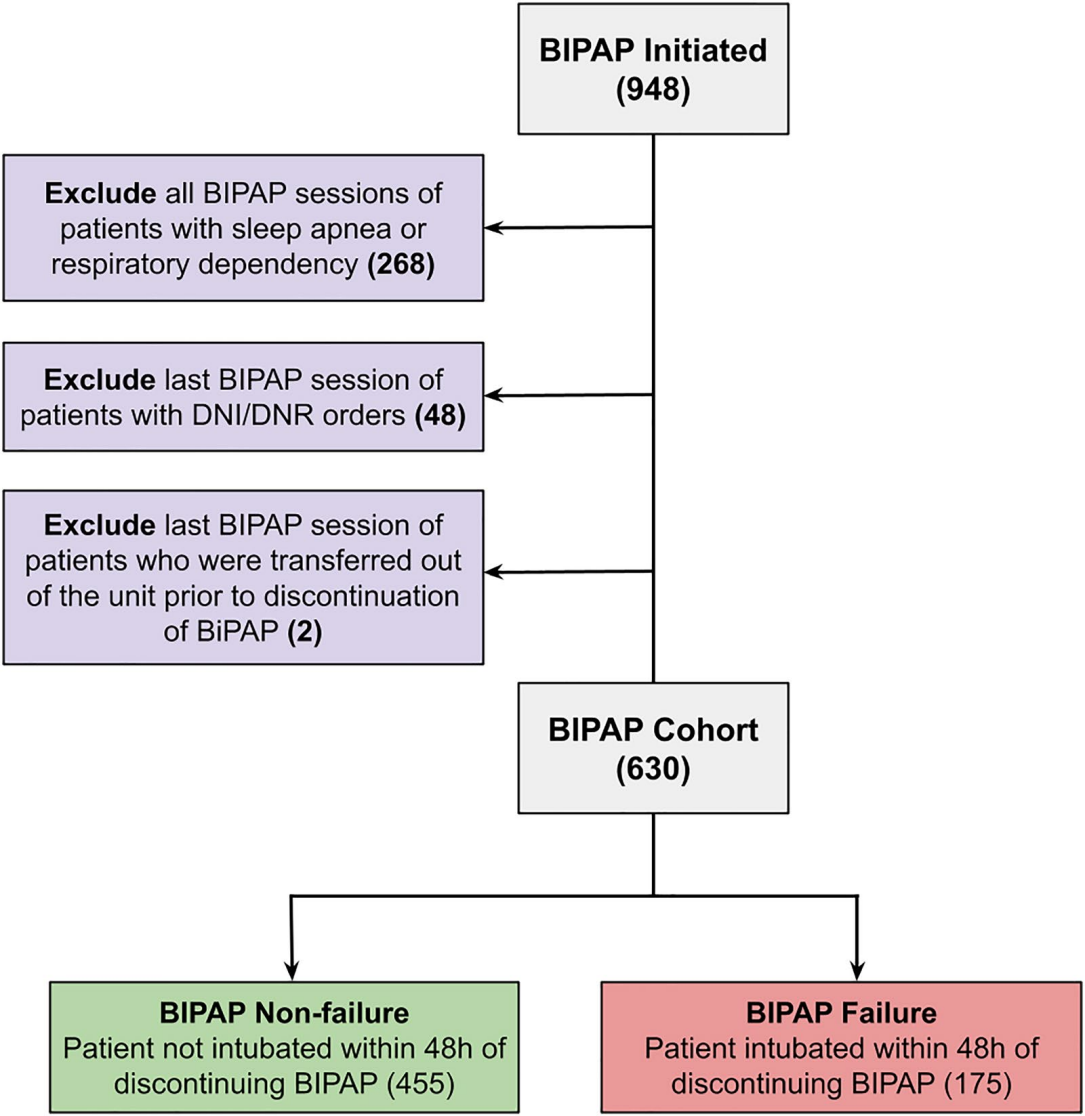
Cohort selection process. The dataset contained 948 instances where BIPAP was initiated. All BIPAP sessions of a patient with a previous diagnosis of respirator/ventilator dependence or sleep apnea were excluded. The last BIPAP session of any patient with a DNI/DNR order or who was transferred out of the unit prior to the BIPAP being discontinued was excluded. BIPAP sessions that resulted in intubation within 48 h of BIPAP discontinuation were considered BIPAP failures.

The inclusion and exclusion criteria resulted in 630 BIPAP sessions, 175 (27.7%) of which met the definition for BIPAP failure: requiring escalation to invasive mechanical ventilation within 48 h of BIPAP termination (Fig. 1). Table 1 shows the characteristics of the entire cohort, partitioned into failures and non-failures. The average age in the two groups (10.7 years in the BIPAP success group and 9.9 years in the BIPAP failure group) were not significantly different. The BIPAP failure group was on BIPAP for a median of 32.8 (9.2–91.3) hours prior to intubation. In the BIPAP failure group, the median time interval between BIPAP termination and mechanical ventilation was 1.7 h (IQR 1.0–2.5 h). The BIPAP failure group had greater PRISM-III scores than the non-failure group. Among the BIPAP sessions lasting at least six hours, the respiratory rate at the 6th hour after BIPAP initiation of the BIPAP failure group was higher than that of the BIPAP success group (median of 31 vs 25 breaths per minute). The average CBG pH in the BIPAP failure group was significantly lower than in the success group. The average SpO_2_/FiO_2_ ratio in the BIPAP failure group, 194, was significantly lower than in the BIPAP success group, 243. The median BIPAP Inspiratory Positive Airway Pressure (IPAP) was 16.0 in both the BIPAP Success (IQR 12.0–18.0) and BIPAP Failure (IQR 14.0–18.5) groups. The median BIPAP Expiratory Positive Airway Pressure (EPAP) was 8.0 in both BIPAP success (IQR 6.0–8.0) and BIPAP failure (IQR 6.0–10.0) groups. When comparing the pediatric complex chronic conditions in both groups (Supplementary Table 4), the BIPAP failure group had higher incidence rates of oncologic (15.4% vs 9.5%), rheumatologic (4.0% vs 1.3%), and metabolic (4.6% vs 1.5%) conditions.

**Table 1:** Demographics and physiologic variables study participants (*P* values from Mann–Whitney U test).

Demographic or characteristic	BIPAP Non-failure (n = 455 Sessions)	BIPAP Failure (n = 175 Sessions)	*P* value
Age (years)	10.7 (5.0–14.5)	9.9 (4.3–13.7)	0.08
Females, n (%)	212 (46.6%)	88 (50.3%)	0.20
Weight (kg): Median (IQR)	29.0 (17.0–45.0)	29.0 (16.0–42.0)	0.42
PRISM III: Median (IQR)	4.0 (1.0–8.0)	7.0 (3.0–11.5)	** < 0.001**
Hours on BIPAP (hours): Median (IQR)	64.0 (25.0–147.2)	32.8 (9.2–91.3)	** < 0.001**
ICU LoS (hours): Median (IQR)	112.0 (55.8–197.8)	328.8 (199.1–560.7)	** < 0.001**
# of Deaths (% of n)	2 (0.4%)	36 (20.6%)	** < 0.001**
**Physiologic variables 6 h after BIPAP initiation (Patients on BIPAP for longer than 6 h only) median (IQR), n**			
Heart Rate, beats per minute	117.0 (98.0–136.0), n = 455	123.0 (104.0–143.7) n = 175	**0.01**
CBG pH (VBG, ABG data in Supplementary Table 4)	7.39 (7.35–7.42) n = 127	7.35 (7.33–7.39) n = 52	** < 0.01**
CBG PCO2 (VBG, ABG data in Supplementary Table 4)	42.0 (36.0–50.5) n = 127	47.0 (39.8–55.0) n = 52	** < 0.01**
SpO_2_, %	99 (98–100) n = 455	98.0 (96–100) n = 175	** < 0.001**
FiO_2_, %	40 (30–45) n = 413	50 (40–70) n = 167	** < 0.001**
Respiratory Rate, breaths per minute	25 (20–33) n = 455	31 (22–40) n = 175	** < 0.001**
Glasgow Coma Score	15 (11–15) n = 445	14 (10–15) n = 164	0.01
S/F Ratio	243 (192–320) n = 311	194 (139–243), n = 135	** < 0.001**
BIPAP Inspiratory Positive Airway Pressure (IPAP)	16.0 (12.0–18.0) n = 438	16 (14–18.5) n = 164	** < 0.01**
BIPAP Expiratory Positive Airway Pressure (EPAP)	8.0 (6.0–8.0) n = 446	8.0 (6.0–10.0) n = 166	** < 0.01**

Significant values are in bold.

### Outcomes in early, intermediate, late BIPAP failure

Table 2 compares some outcomes of patients with early (< 6 h on BIPAP before intubation), intermediate (6–24 h), or late (> 24 h) BIPAP failure. Patients with late BIPAP failure had significantly fewer 28-VFD IMV BIPAP (13.40 [0.68–20.96]) than the early (16.58 [7.37–23.06]) and intermediate (20.14 [6.91–23.76]) BIPAP failure groups. The late BIPAP failure group had significantly higher ICU LOS, post-extubation BIPAP days, and a higher proportion of patients requiring post-extubation BIPAP compared to those in the early and intermediate BIPAP failure groups.

**Table 2:** Outcomes parsed by hours between BIPAP initiation and BIPAP failure (*P* values from Kruskal–Wallis H-test).

Characteristic / Outcome	Early failure < 6 h (33)	Intermediate failure 6–24 h (63)	Late failure > 24 h (80)	*P* Value
28-VFD IMV (Q1-Q3)	21.1 (9.3–24.4)	21.8 (8.8–24.9)	22.5 (8.5–27.7)	0.29
28-VFD IMV BIPAP, median (Q1–Q3)	16.6 (7.4–23.1)	20.1 (6.9–23.8)	13.4 (0.7–21.0)	**0.04**
Patients requiring post-extubation BIPAP n (%)	16 (48.5%)	28 (44.4%)	53 (67.1%)	**0.02**
Post-extubation BIPAP Days (Q1–Q3) (%)	0.0 (0.0–10.1)	0.0 (0.0–2.9)	3.5 (0.0–9.8)	** < 0.01**
ICU LOS Hours, median (Q1–Q3)	295.6 (185.3–433.7)	316.3 (135.3–546.0)	385.0 (277.5–586.2)	** < 0.01**
PICU Mortality n (%)	6 (18.2%)	11 (17.5%)	19 (24.1%)	0.59

Significant values are in bold.

### Model performance: continuous predictions over time on BIPAP

**Figure 2:**
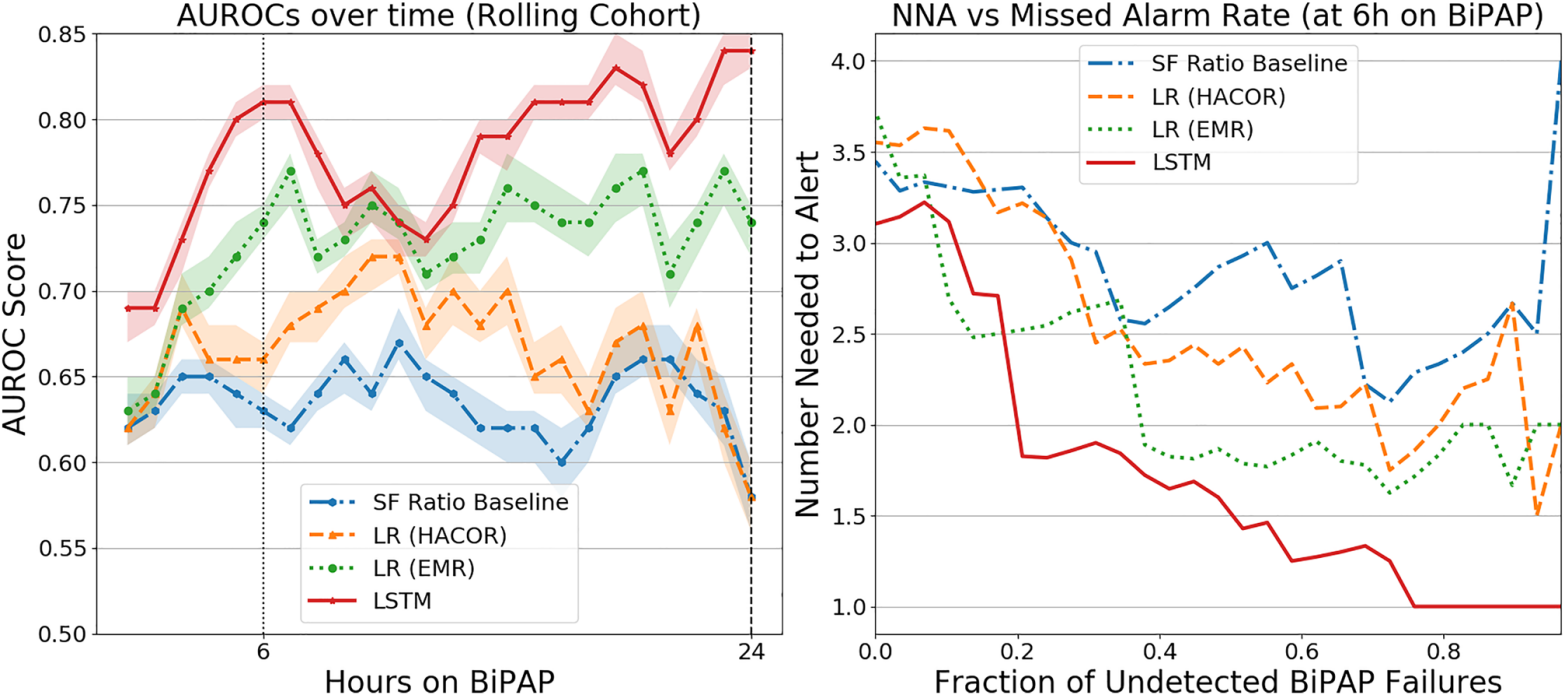
Model performance. (Left) Hourly AUROC values from LSTM-RNN, LR models, and S/F ratio for predicting BIPAP failure. The rolling cohort shows each model’s *n*-hour AUROC for BIPAP episodes with at least *n*-hours of BIPAP. (Right) Number needed to alert plotted as a function of missed alarm rates for predictions at 6 h after BIPAP initiation, capturing intermediate and late BIPAP failures.

**Figure 3:**
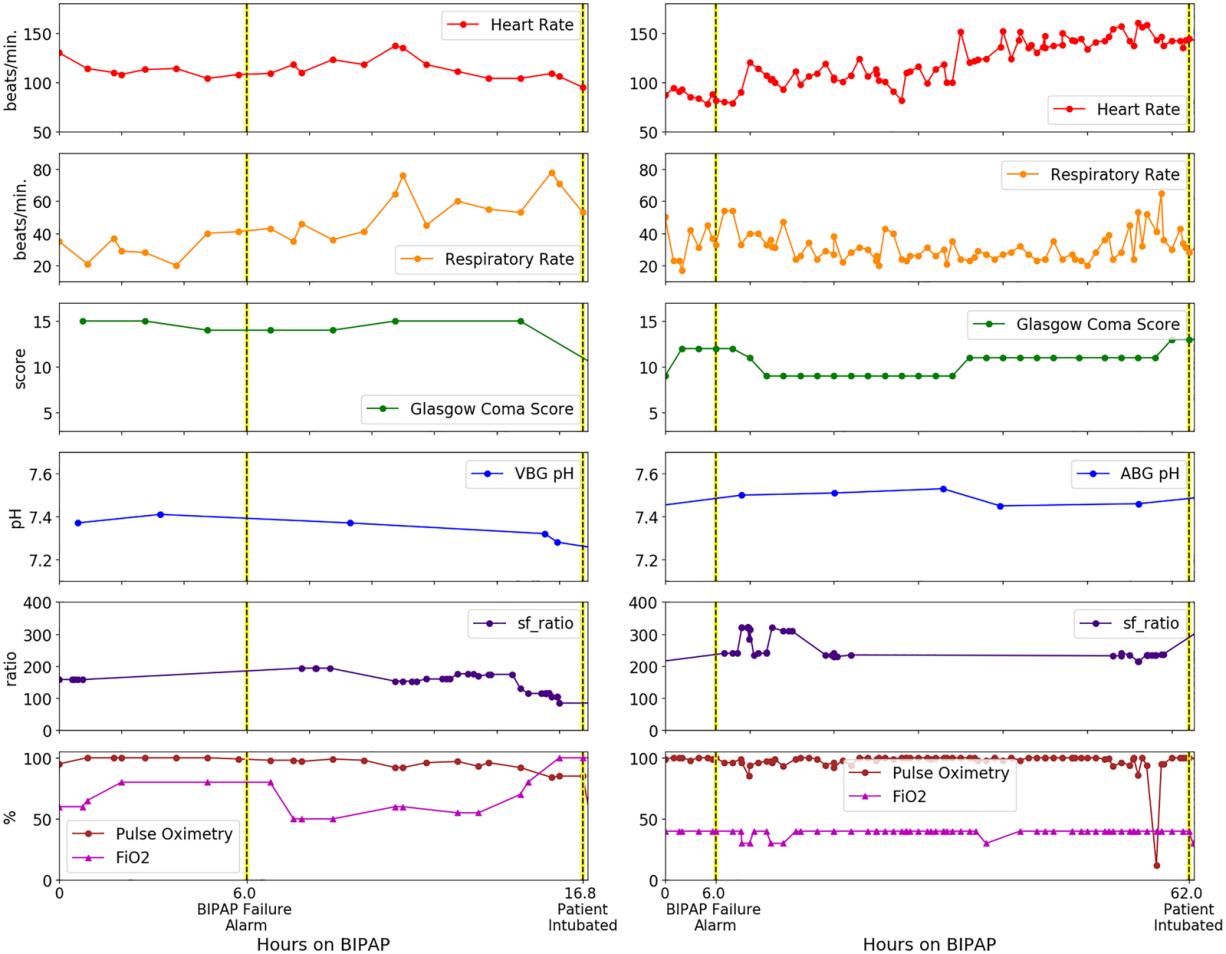
Examples of clinical utility of LSTM-RNN prediction in study patients. (Left) 16-year-old male with Acute Myelocytic Leukemia was admitted for septic shock and ARDS, intubated 17 h after BIPAP initiated. (Right) 3-year-old with hypoxic ischemic encephalopathy, epilepsy, metabolic alkalosis, and malnutrition admitted for respiratory failure, intubated 62 h after BIPAP initiation. In both cases, LSTM-RNN model predicted BIPAP failure at 6-h mark using the threshold of NNA = 1.

At all assessment times, the LSTM-RNN model discriminated better between BIPAP failure and non-failure than the other three models (Fig. 2, left). Counts of failures and non-failures at each assessment hour are in Supplementary Table 5. In particular, the LSTM-RNN model had the highest discrimination both at hour 6, AUROC of 0.81 (0.80, 0.82), and hour 24, 0.84 (0.83, 0.85) (Supplementary Table 6). Further, the LSTM-RNN model performed better than the other models in hypoxemic patients (S/F Ratio ≤ 264) with an AUROC 0.88 (0.88, 0.89) at hour 6 and 0.86 (0.85, 0.87) at hour 24 (Supplementary Table 6). Only the LSTM-RNN model performed better in hypoxemic patients than in the general cohort. The LSTM model demonstrated a consistent trend of lower NNA than the other models across sensitivity (Fig. 2, right). When operating at NNA = 2, the LSTM-RNN model identified nearly 80% of BIPAP failures within 6 h. For NNA = 1 (i.e., no false alarms) within 6 h of BIPAP initiation, the LSTM-RNN identified almost 25% of BIPAP failures (7 BIPAP episodes). All other models at this sensitivity had at least one false alarm. Figure 3 shows two BIPAP episodes where the LSTM-RNN predicted BIPAP failure after 6 h using the threshold of NNA = 1; they were intubated 11 and 56 h after the alarm. The other five BIPAP episodes correctly predicted to fail (with NNA = 1) were intubated 5, 21, 44, 51, and 94 h after the alarm.

## Discussion

Identifying children likely to fail BIPAP is important for several reasons. First, there is increasing concern that patients on NIV with high respiratory effort may exacerbate injury to their own lungs, a term deemed patient self-inflicted lung injury^12^. The mechanisms of P-SILI have been well described and include lung stress with high transpulmonary pressure, lung strain with high tidal volume relative to end-expiratory lung volume, atelectrauma, and pendeluft^12–18^. If patients are breathing with these injurious patterns on BIPAP, then longer duration of exposure to BIPAP without reducing respiratory work with intubation, sedation, and or neuromuscular blockade may lead to lung injury progression. Importantly, our findings corroborate recent data that pediatric ARDS patients receiving pre-intubation BIPAP for greater than or equal to 24 h have longer lengths of PICU and hospital stay and higher 28- and 90-day mortality compared to ARDS patients who were either intubated primarily or on BIPAP for less than 24 h^6^. In addition, children who are intubated after failing BIPAP have higher rates of complications during intubation such as desaturation, prolonged hypoxemia, and even cardiac arrest^6–10,21^. This evidence supports the hypothesis that timely identification and intervention in children likely to fail BIPAP may prevent complications. The HACOR scale was developed to predict NIV failure in hypoxemic adults, but there are no decision support tools (of which the authors are aware) for BIPAP failure in children.

This study demonstrated that a deep learning model (LSTM-RNN) using readily available EMR data could identify children at risk for BIPAP failure. In the general cohort, the LSTM-RNN model achieved higher AUROCs than the two logistic regression models (one using the same inputs as the LSTM-RNN model and another using five physiologic variables predictive of NIV failure) and the S/F ratio at every assessment hour. In hypoxemic patients (S/F ratio < 264), a group of particular interest^23,24,34^ because their risks of P-SILI are higher, the LSTM-RNN model also had AUROC than all other models. Interestingly, the LSTM-RNN model had higher AUROC in the hypoxemic group than in the general cohort, which was not the case for the other models.

It is important to note that the predictions are predicated on the clinical intervention deemed appropriate by the care team. The performance measures the ability of the models to predict BIPAP failure given that the clinical team determined it was appropriate to keep the child on BIPAP. A model with a 0.5 AUROC is equivalent to a clinical team making a decision to keep a child on BIPAP; an AUROC above 0.5 indicates that a model has extracted new information, potentially valuable to the clinical team, about BIPAP failure.

Model performance at hour 6 is noteworthy because a large proportion of children who ultimately fail BIPAP and get intubated do so more than 6 h after BIPAP initiation. Because interventions such as intubation have risks, a diagnostic tool in this domain must have a modest or low false alarm rate. The NNA vs detection plot in Fig. 2 demonstrates the advantage of the LSTM-RNN model over the other models. Nearly 80% of the BIPAP failures can be identified within 6 h, with an NNA of 2. At an NNA of 1 (i.e., no false alarms), the LSTM-RNN identified almost 25% of the BIPAP failures with a median time to failure of 44 h from the alarm. The LSTM-RNN can prompt clinicians to take a closer look at these high-risk patients to determine the best course of action, which may include intubation or adjusting BIPAP settings.

Many clinicians regularly follow variables such as the S/F ratio to gauge clinical response to BIPAP, which in these experiments had some prognostic relevance, but had more false alarms than the LSTM-RNN model. The LR models learned meaningful relationships among all available EMR variables and the target outcome to make better predictions. The LSTM-RNN model’s ability to include time dependencies and multiple, unselected variables resulted in superior performance in finding and understanding relationships between the patient’s dynamic clinical state and the target outcome of BIPAP failure. Furthermore, the results demonstrated that the LSTM-RNN model can learn over time as the patient’s condition changes to continuously improve its prediction of BIPAP failure. Model performance at 6 and 24 h were highlighted, but in fact the model can generate a prediction as soon as enough data is available (i.e., even within the first hour). This may be important in future applications to identify patients failing within the first hour of BIPAP. The results of this proof-of-concept study demonstrate the feasibility of analyzing critical care data with advanced ML methods to provide clinical decision support. A tool could be integrated into the clinical workflow, either as part of bedside monitoring or a webtool easily accessed by clinicians to obtain dynamic predictions on important patient outcomes. The actual design and implementation of such tools require careful understanding of many different areas well outside the scope of this study^38,39^.

This study had a few important limitations due to its single center, retrospective nature. BIPAP and intubation practices, including the selection of airway pressure and supplemental oxygen settings, may be different at our institution compared to others, which may affect generalizability. Additionally, to generate the sample size needed for the analysis, we used data spanning 10 years, and there may have been important changes to practice with respect to non-invasive ventilation for respiratory failure. We did not have information on the type of BIPAP interface used (oral versus nasal). Another limitation was that the RNN was trained for a target outcome of BIPAP failure that was subjectively biased as a complex human bedside clinician decision. Finally, we excluded patients who may be on home BIPAP (i.e., excluding patients with a diagnosis of obstructive sleep apnea).

## Conclusions

A machine learning model using electronic health record data from children on BIPAP can identify children with high likelihood of failing BIPAP, with a relatively modest false alarm rate. We suggest external validation of this model in additional ICUs to test its generalizability, followed by a clinical trial to determine if it results in improved outcomes for children with acute respiratory failure on BIPAP.

## Supplementary Information

Supplementary Information.

## Data Availability

The data comes from electronic medical records from Children’s Hospital Los Angeles, and it is not publicly available as it contains sensitive information of patients.

## Code availability

The code written to process and analyze the data can be made available upon request to the corresponding author.
