# Supplementary material for: Development of a deep learning model that predicts Bi-level positive airway pressure failure
Source: Sci Rep. 2022 May 26;12:8907. doi: 10.1038/s41598-022-12984-x (PMC9135753; doi:10.1038/s41598-022-12984-x)
Supplement: Supplementary file 1 — Supplementary Information. [file 41598_2022_12984_MOESM1_ESM.docx]

**Supplementary Tables and Figures Legend**

Supplementary Table 1. EMR variables (demographics, vitals and labs) in patient episode matrix. Demographics such as gender and race/ethnicity were encoded as binary variables.

Supplementary Table 2. EMR Variables (drugs and interventions) in patient episode matrix

Supplementary Table 3. Hyperparameters of RNN

Supplementary Table 4. Complex Chronic Conditions and ABG, VBG pH and CO2 of study participants

Supplementary Table 5. Failure to Non-Failure counts at each plotted hour in Figure 2 for the rolling cohort

Supplementary Table 6. AUROC scores for the holdout test set and parsed by S/F Ratio threshold indicating a hypoxemic state at the 6th and 24th hour.

Supplementary Figure 1. Examples of ventilator-free days calculation.

Supplementary Figure 2. S/F Ratio Imputation for Non-Computable Timesteps. Timesteps where SpO_2_ fell outside of S/F Ratio’s computable range were handled by forward-filling and then imputed based on previously recorded SpO_2_ values to make the data amenable to machine learning and representative of the patient's state. A child whose SpO_2_ fell below 80% while their S/F ratio was not computable was considered poorly oxygenated at the time. Therefore, their S/F ratio at these timesteps were imputed with a low value, the bottom 25th percentile of S/F Ratios from the train partition (174.5), to proxy a poorly oxygenated state. Similarly, a child whose SpO_2_ remained above 98% while their S/F ratio was not computable was considered well oxygenated at the time. In this case, their S/F ratio at these timesteps were imputed with a high value, the 75th percentile of S/F ratios from the training partition (316.7), to proxy a well oxygenated state.

**Supplementary Table 1. EMR variables (demographics, vitals and labs) in patient episode matrix. Demographics such as gender and race/ethnicity were encoded as binary variables.**

| **Demographics and Vitals** | | | |
| --- | --- | --- | --- |
| Abdominal Assessment Distended | Abdominal Assessment Firm | Abdominal Assessment Flat | Abdominal Assessment Round |
| Abdominal Assessment Soft | Abdominal Assessment Tender | Abdominal Girth | Activity Level |
| Age | Behavioral Assessment Active | Behavioral Assessment Agitation | Behavioral Assessment Awake Quiet |
| Behavioral Assessment Disrupted Sleep | Behavioral Assessment Drowsy | Behavioral Assessment Grimaces | Behavioral Assessment Occasional Cry |
| Behavioral Assessment Restless | Behavioral Assessment Sedated | Behavioral Assessment Sleeping | Behavioral Assessment Verbal |
| Bowel Sounds | Breath Sounds Clear | Breath Sounds Coarse | Breath Sounds Coarse Crackles |
| Breath Sounds Diffuse | Breath Sounds Diminished | Breath Sounds Fine Crackles | Breath Sounds Moist Crackles |
| Breath Sounds Rhonchi | Breath Sounds Scattered | Breath Sounds Squeaks | Breath Sounds Wheezing |
| CAPD Score | Capillary Refill Delayed | Capillary Refill Rate | Central Venous Pressure |
| Cough Present | Delta T | Diastolic Blood Pressure | EtCO_2_ |
| Extremity Temperature Level | Eye Response Level | FLACC Pain Activity | FLACC Pain Consolability |
| FLACC Pain Cry | FLACC Pain Face | FLACC Pain Intensity | FLACC Pain Legs |
| Gag Present | Glasgow Coma Score | Heart Rate | Heart Sounds Normal |
| Height | Inotrope Score | Left Pupil Size After Light | Left Pupil Size Before Light |
| Left Pupillary Response Level | Level of Consciousness | Lip Moisture Level | Mean Arterial Pressure |
| Motor Response Level | Nasal Flaring Level | Nutrition Level | PaO_2_ To FiO_2_ |
| Peripheral Intravenous Line Site | Potential for Pain Burn | Potential for Pain Chest Tube | Potential For Pain Disease Process |
| Potential for Pain Invasive Line | Potential for Pain Postop | Potential for Pain Postop24Hr | Pulse Oximetry |
| Quality of Pain Level | Race Asian/Indian/Pacific Islander | Race Black or African American | Race Hispanic Or Latino |
| Race Unknown | Race White | Respiratory Effort Level | Respiratory Rate |
| Right Pupil Size After Light | Right Pupil Size Before Light | Right Pupillary Response Level | SBS Score |
| Sedation Scale Level | Sex F | Sex M | S/F Ratio |
| Side Rails | Skin Integrity Dry | Skin Turgor Edema | Skin Turgor Turgor |
| Systolic Blood Pressure | Temperature | Verbal Response Level | Wat1 Total |
| Weight |  |  |  |
| **Labs** | | | |
| ABG Base Excess | ABG FiO_2_ | ABG HCO_3_ | ABG O2 Sat |
| ABG PCO_2_ | ABG pH | ABG PO_2_ | ABG TCO_2_ |
| Albumin Level | Alkaline Phosphatase | ALT | Amylase |
| AST | B-Type Natriuretic Peptide | Bands % | Basophils % |
| Bicarbonate Serum | Bilirubin Conjugated | Bilirubin Total | Bilirubin Unconjugated |
| BUN | C-Reactive Protein | Calcium Ionized | Calcium Total |
| Cardiac Rhythm Normal Sinus | Cardiac Rhythm Sinus Bradycardia | Cardiac Rhythm Sinus Tachycardia | CBG Base Excess |
| CBG FiO_2_ | CBG HCO_3_ | CBG O_2_ Sat | CBG PCO_2_ |
| CBG Ph | CBG PO_2_ | CBG TCO_2_ | Chloride |
| Creatinine | Culture Blood | Culture Respiratory | Culture Urine |
| Eosinophils % | Fibrinogen | Glucose | Hematocrit |
| Hemoglobin | Influenza Lab | INR | Lactate |
| Lactate Dehydrogenase Blood | Lipase | Lymphocyte % | Macrocytes |
| Magnesium Level | MCH | MCHC | MCV |
| Metamyelocytes % | Monocytes % | Myelocytes % | Neutrophils % |
| Phosphorus Level | Platelet Count | Potassium | Protein Total |
| PT | PTT | RBC Blood | RDW |
| Reticulocyte Count | Schistocytes | Sodium | Triglycerides |
| VBG Base Excess | VBG Fio2 | VBG HCO_3_ | VBG O_2_ Sat |
| VBG PCO_2_ | VBG pH | VBG PO_2_ | VBG TCO_2_ |
| White Blood Cell Count |  |  |  |

**Supplementary Table 2. EMR Variables (drugs and interventions) in patient episode matrix**

| **Drugs** | | | |
| --- | --- | --- | --- |
| Acetaminophen Inter | Albumin Inter | Albuterol Inter | Alteplase Inter |
| Aminophylline Cont | Aminophylline Inter | Ampicillin/Sulbactam Inter | Azithromycin Inter |
| Baclofen Inter | Budesonide Inter | Calcium Chloride Inter | Cefazolin Inter |
| Cefepime Inter | Ceftazidime Inter | Ceftriaxone Inter | Chlorothiazide Inter |
| Clindamycin Inter | Clonazepam Inter | Clonidine HCl Inter | Dexamethasone Inter |
| Dexmedetomidine Cont | Diazepam Inter | Diphenhydramine HCl Inter | Dopamine Cont |
| Famotidine Inter | Fentanyl Cont | Fentanyl Inter | Fluconazole Inter |
| Furosemide Cont | Furosemide Inter | Glycopyrrolate Inter | Heparin Flush Inter |
| Hydrocortisone Inter | Hydromorphone Inter | Ibuprofen Inter | Insulin Inter |
| Ipratropium Bromide Inter | Isradipine Inter | Ketamine Inter | Ketorolac Inter |
| Lactobacillus Inter | Lansoprazole Inter | Levalbuterol Inter | Levetiracetam Inter |
| Levocarnitine Inter | Lorazepam Inter | Magnesium Sulfate Inter | Meropenem Inter |
| Methylprednisolone Inter | Metoclopramide Inter | Midazolam Hcl Inter | Morphine Inter |
| Ondansetron Inter | Pantoprazole Inter | Phenobarbital Inter | Piperacillin/Tazobactam Inter |
| Potassium Chloride Inter | Ranitidine Inter | Rocuronium Inter | Sodium Bicarbonate Inter |
| Sodium Chloride Inter | Trimethoprim/Sulfamethoxazole Inter | Valproic Acid Inter | Vancomycin Inter |
| Vitamin K Inter |  |  |  |
| **Interventions** | | | |
| Arterial Line Site | BiPAP Num | BiPAP Session Num | Central Venous Line Site |
| Chest X Ray | Comfort Response Level | Diversional Activity Music | Diversional Activity Play |
| Diversional Activity Toys | Diversional Activity TV | EPAP | Ever Intubated |
| FiO_2_ | Gastrostomy Tube Location | Inspiratory Time | IPAP |
| Mean Airway Pressure | Mechanical Ventilation Mode | Multidisciplinary Team Present | BIPAP Mode |
| BiPAP Set Rate | Nurse Activity Level Completed Ambulate | Nurse Activity Level Completed Bedrest | Nurse Activity Level Completed Chair |
| Nurse Activity Level Completed Commode | Nurse Activity Level Completed Held | Nurse Activity Level Completed Logroll | Nurse Activity Level Completed Microturns |
| Nurse Activity Level Completed Out of bed | Nurse Activity Level Completed Repositioned | Nurse Activity Level Completed Turn | Nurse Activity Level Completed Tv |
| O_2_ Flow Rate | On BiPAP | Oxygen Mode Level | Peak Inspiratory Pressure |
| PEEP | Pharmacological Comfort Measures Given | Position Support Given | Position Tolerance Level |
| Pressure Support | Range Of Motion Assistance Type | Sedation Intervention Level | Sedation Response Level |
| Tidal Volume Delivered | Tidal Volume Expiratory | Tidal Volume Inspiratory | Ventilator Rate |
| Visitor Mood Level | Visitor Present Aunt | Visitor Present Father | Visitor Present Friend |
| Visitor Present Grandfather | Visitor Present Grandmother | Visitor Present Mother | Visitor Present Sibling |
| Visitor Present Uncle | Volume Tidal |  |  |

**Supplementary Table 3. Hyperparameters of RNN-LSTM Model**

| **Hyperparameter** | **Value** |
| --- | --- |
| Hidden Units in LSTM Layer | 256 |
| Batch Size | 16 |
| Learning Rate | 1e-3 |
| Loss | RMSProp |
| Optimizer | RMSProp |
| Dropout | Binary Cross Entropy |
| Regularizer | 1e-3 |
| Output Activation | Sigmoid |

**Supplementary Table 4. Complex Chronic Conditions and ABG, VBG data of study participants**

| *Complex Chronic Conditions, n (%)* | BIPAP Non-failure  (n = 455 Sessions) | BIPAP Failure  (n = 175 Sessions) | P-value |
| --- | --- | --- | --- |
| Cardiovascular | 12 (2.6%) | 1 (0.6%) | 0.05 |
| Genetic | 14 (3.1%) | 4 (2.3%) | 0.30 |
| Gastrointestinal | 6 (1.3%) | 1 (0.6%) | 0.21 |
| Oncologic | 43 (9.5%) | 27 (15.4%) | **0.02** |
| Neurologic | 25 (5.5%) | 8 (4.6%) | 0.32 |
| Rheumatologic | 6 (1.3%) | 7 (4.0%) | **0.02** |
| Metabolic | 7 (1.5%) | 8 (4.6%) | **0.01** |
| Endocrinologic | 24 (5.3%) | 13 (7.4%) | 0.15 |
| Immunologic | 6 (1.3%) | 3 (1.7%) | 0.35 |
| Respiratory | 52 (11.4%) | 19 (10.9%) | 0.42 |
| Hematologic | 13 (2.9%) | 7 (4.0%) | 0.23 |
| Psychiatric | 0, (0.0%) | 0, (0.0%) | n/a |
| *ABG pH and VBG pH 6 hours after BIPAP initiation (Patients on BIPAP for longer than 6 hours only) median (IQR), n* |  |  |  |
| ABG pH | 7.37 (7.34-7.40)  n=31 | 7.37 (7.32-7.39) n=10 | 0.48 |
| VBG pH | 7.35 (7.32-7.40)  n=69 | 7.36 (7.27-7.41) n=48 | 0.16 |
| ABG PCO2 | 38.0 (33.0 – 47.5) n=31 | 45.5 (31.8 – 61.2) n=10 | 0.19 |
| VBG PCO2 | 48.0 (42.0 – 60.0) n=69 | 48.0 (40.0 – 55.2) n=48 | 0.29 |

**Supplementary Table 5. Failure to Non-Failure counts at each plotted hour in Figure 2 for the rolling cohort**

| \| **Hours on BiPAP** \| **Failure:Non-Failure** \| \| --- \| --- \| \| 1 \| 36:91 \| \| 2 \| 35:86 \| \| 3 \| 33:85 \| \| 4 \| 30:82 \| \| 5 \| 29:82 \| \| 6 \| 29:80 \| \| 7 \| 27:78 \| \| 8 \| 26:76 \| \| 9 \| 25:72 \| \| 10 \| 25:69 \| \| 11 \| 24:66 \| \| 12 \| 24:65 \| | \| **Hours on BiPAP** \| **Failure:Non-Failure** \| \| --- \| --- \| \| 13 \| 23:64 \| \| 14 \| 22:64 \| \| 15 \| 21:64 \| \| 16 \| 20:64 \| \| 17 \| 19:63 \| \| 18 \| 19:62 \| \| 19 \| 19:61 \| \| 20 \| 19:59 \| \| 21 \| 17:57 \| \| 22 \| 17:55 \| \| 23 \| 17:52 \| \| 24 \| 16:52 \| |
| --- | --- | --- | --- | --- | --- | --- | --- | --- | --- | --- | --- | --- | --- | --- | --- | --- | --- | --- | --- | --- | --- | --- | --- | --- | --- | --- | --- | --- | --- | --- | --- | --- | --- | --- | --- | --- | --- | --- | --- | --- | --- | --- | --- | --- | --- | --- | --- | --- | --- | --- | --- | --- | --- |

**Supplementary Table 6. AUROC scores for the holdout test set and parsed by S/F Ratio threshold indicating a hypoxemic state at the 6th and 24th hour.**

|  |  | **AUROC Score (For Rolling Cohort)** | | | | |
| --- | --- | --- | --- | --- | --- | --- |
| **Cohort** | **Pred Time** | **Failure: Non-Failure** | **S/F Ratio Baseline** | **LR_HACOR_** | **LR_EMR_** | **LSTM** |
| **All** | 6 | 29:80 | 0.63  (0.62, 0.64) | 0.66  (0.64, 0.67) | 0.74  (0.73, 0.75) | 0.81  (0.80, 0.82) |
|  | 24 | 16:52 | 0.58  (0.56, 0.60) | 0.58  (0.56, 0.60) | 0.74  (0.72, 0.75) | 0.84  (0.83, 0.85) |
| **S/F Ratio**  **< 264** | 6 | 10:22 | 0.58  (0.56, 0.59) | 0.50  (0.49, 0.52) | 0.73  (0.72, 0.74) | 0.88  (0.88, 0.89) |
|  | 24 | 8:18 | 0.49  (0.48, 0.51) | 0.42  (0.40, 0.44) | 0.59  (0.58, 0.61) | 0.86  (0.85, 0.87) |


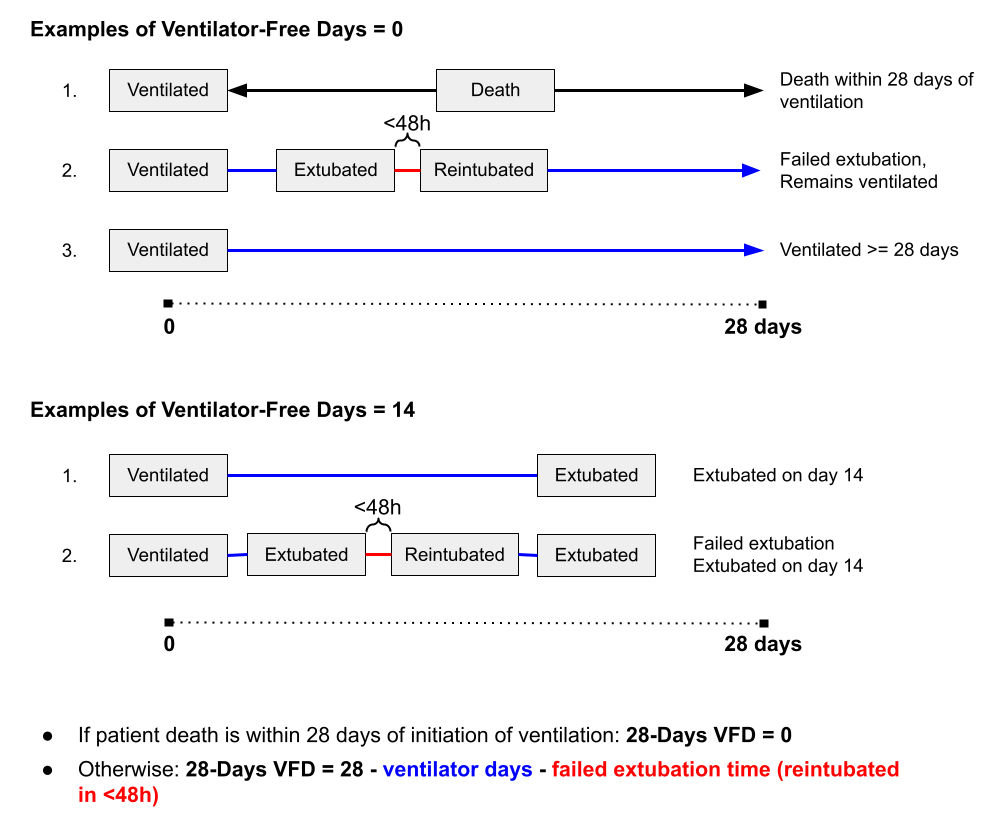


**Supplementary Figure 1. Examples of ventilator-free days calculation.**


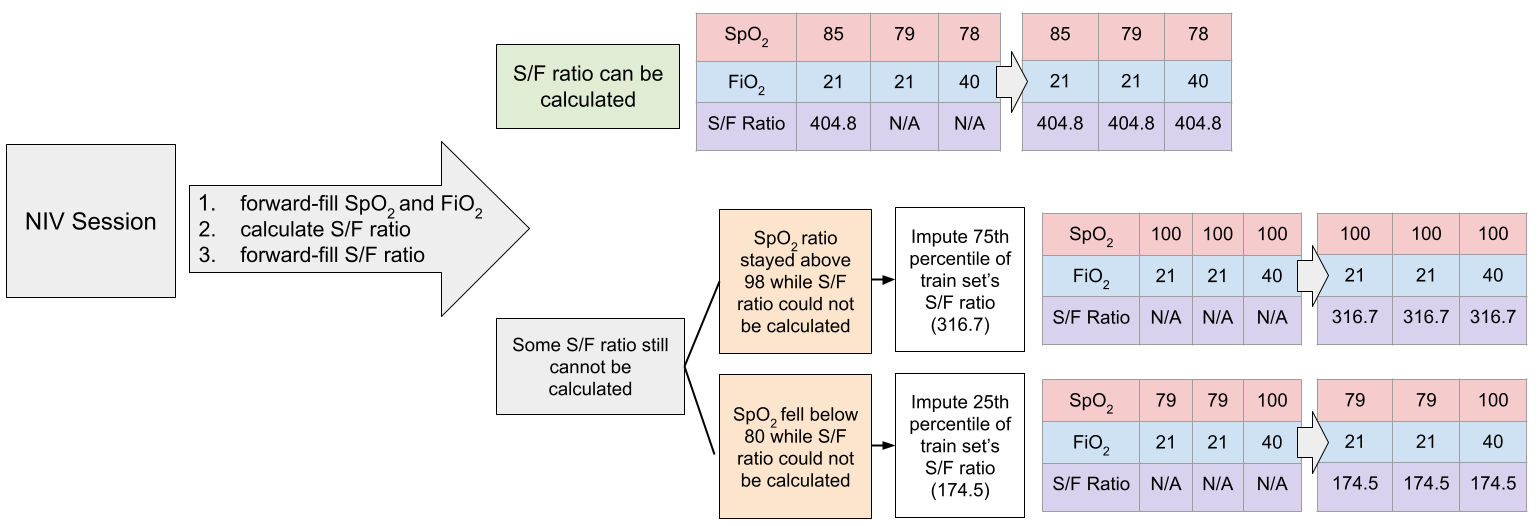

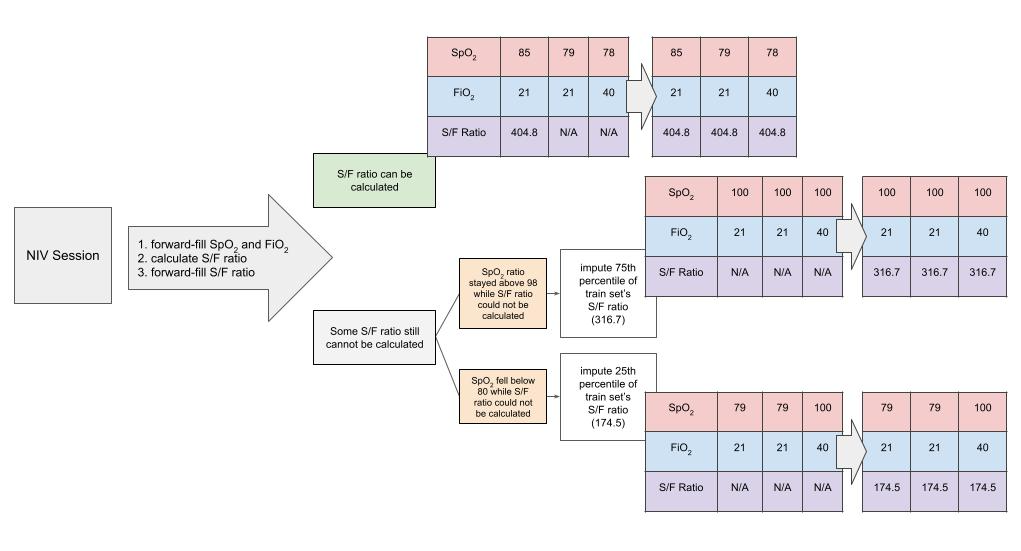


**Supplementary Figure 2. S/F Ratio Imputation for Non-Computable Timesteps. Timesteps where SpO_2_ fell outside of S/F Ratio’s computable range were handled by forward-filling and then imputed based on previously recorded SpO_2_ values to make the data amenable to machine learning and representative of the patient's state. A child whose SpO_2_ fell below 80% while their S/F ratio was not computable was considered poorly oxygenated at the time. Therefore, their S/F ratio at these timesteps were imputed with a low value, the bottom 25th percentile of S/F Ratios from the train partition (174.5), to proxy a poorly oxygenated state. Similarly, a child whose SpO_2_ remained above 98% while their S/F ratio was not computable was considered well oxygenated at the time. In this case, their S/F ratio at these timesteps were imputed with a high value, the 75th percentile of S/F ratios from the training partition (316.7), to proxy a well oxygenated state.**

**
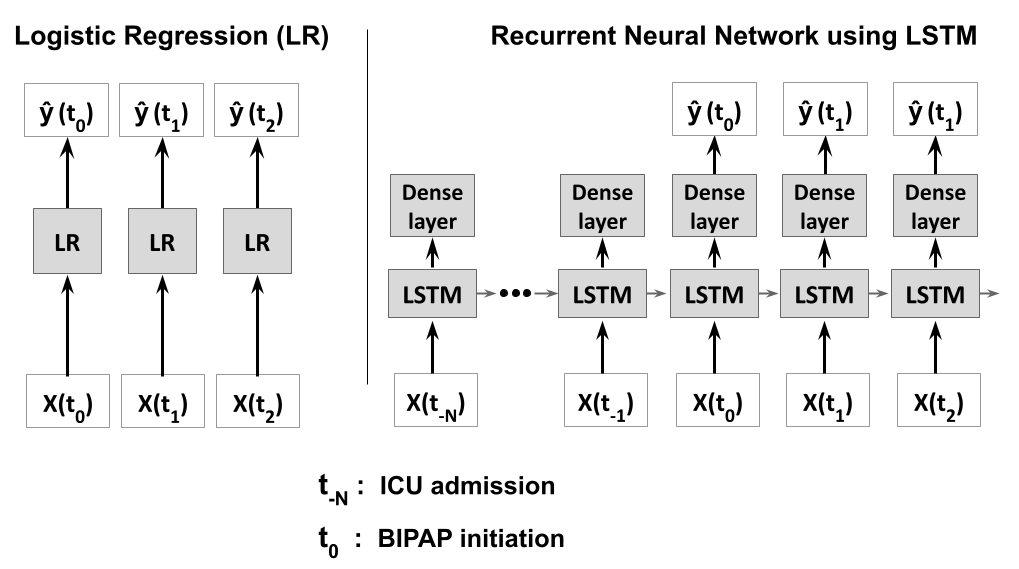
**

**Supplementary Figure 3. Details of Logistic Regression and Recurrent Neural Network Models. When a new set of measurements is available at time t_n_, the data pre-processor generates from it the input vector, x(t_n_). It is passed into the Recurrent Neural Network (RNN) and Logistic Regression (LR) models, which make a prediction, ŷ((t_n_). While the LR's output is based solely on the immediate observation, the RNN integrates the immediate observation with information from previous observations to make its prediction.**
